# Exploring the relationships among language input-output, vocabulary knowledge, and English Reading development of emergent bilinguals in dual language immersion programs

**DOI:** 10.1080/13670050.2025.2546423

**Published:** 2025-08-19

**Authors:** Becky H. Huang, Ji-Young Choi

**Affiliations:** a Department of Teaching and Learning & Crane Center for Early Childhood Research and Policy, The Ohio State University, Columbus, OH, USA;; b Department of Human Sciences & Crane Center for Early Childhood Research and Policy, The Ohio State University, Columbus, OH, USA

**Keywords:** Emergent bilinguals, dual language immersion programs, language input-output, vocabulary, reading, cross-linguistic transfers

## Abstract

Learning to read in a second language (L2) poses unique challenges for emergent bilingual (EB) children. This study examines whether Spanish-English EB children’s vocabulary skills in Spanish and English mediate the relationship between their language input-output and English (L2) reading skills. The sample included 338 Latina/o EB children in first and third grades enrolled in dual-language immersion (DLI) programs in the United States. Results from the multivariate regression model revealed differences in language input-output at home, with third graders reported using more English than first graders. However, no grade-level differences were observed in language input-output at school in both languages, underscoring the important role of DLI programs in maintaining EB children’s first language (L1). The findings also revealed the robust contributions of both L1 and L2 input-output to English vocabulary, which was positively associated with advanced English reading outcomes. When examining the indirect effects, EB children’s English use at home and Spanish use at school significantly predicted their English reading outcomes through their advanced English vocabulary skills. These findings demonstrated the additive role of home language use in supporting L2 reading development. The results also underscore the importance of fostering EBs’ vocabulary in both languages to enhance their English reading.

## Introduction

Learning to read in a second language (L2) presents unique challenges for bilingual learners, particularly those who are still emergent learners of the L2. Unlike their monolingual peers, who typically have established foundational oral language skills by the time they begin learning to read, emergent bilingual (EB) learners face a dual burden: learning the L2 oral language and developing reading skills in that language.

According to the *Simple View of Reading (SVR)*, reading comprehension is predicted by the combination of decoding and language comprehension (Hoover and Gough 1990). Decoding refers to the ability to recognize letters or words, and strong decoding skills allow readers to focus on comprehending text rather than identifying words. Language comprehension, on the other hand, involves comprehension of oral language components, such as vocabulary and syntactic knowledge (Language and Reading Research Consortium [LARRC] 2015). Among the components of language comprehension, vocabulary is one of the strongest predictors of reading comprehension. Readers with well-developed mental representations of words that include detailed information about each word’s phonology, orthography, and meaning can efficiently recognize them, freeing cognitive resources for comprehension (Perfetti and Hart 2001; Quinn et al. 2015). A positive relationship between vocabulary and reading skills has also been consistently demonstrated in research on bilingual children. Notably, studies comparing monolinguals and EBs learning to read in L2 English have found stronger language-reading correlations for EBs than for their monolingual peers (Geva and Farnia 2012). The literature on EBs’ vocabulary development has further identified language input-output as key predictors of vocabulary growth (Choi, Jeon, and Arabzadehjafari 2024; Hoff et al. 2012; Paradis, Genesee, and Crago 2011; Ribot, Hoff, and Burridge 2018). Taken together, these studies indicate the potential mediation role of vocabulary development on the association between language input-output and reading skills.

The current literature provides a solid understanding of the associations between language input-output and vocabulary development, as well as the relationship between vocabulary and reading skills. However, to our knowledge, no study to date has examined how these three constructs are interconnected. This study aims to address this gap by studying vocabulary skills as a potential pathway through which language input-output influences L2 reading development.

### Language input-output and vocabulary development

Language input generally refers to the linguistic stimuli children are exposed to through mediums such as conversations with others, books, or media. Language output involves the opportunities children have to actively produce oral or written language. While input provides the linguistic stimuli necessary for comprehension and language acquisition (Hoff 2013; Rowe 2012), output allows children to practice, refine, and internalize their developing language skills (Swain 1985, 2005).

In child language research, language input is typically conceptualized in terms of its quantity and quality. Quantity refers to the total amount of words or utterances in child-directed speech whereas quality encompasses multiple dimensions, including linguistic, conceptual, and interactive features (Rowe and Snow 2020). Although both input quantity and quality are associated with child language outcomes (Huttenlocher et al. 2010; Rowe 2012), input quality generally shows stronger predictive power than quantity. In a longitudinal study, Rowe (2012) examined the influence of input quantity and quality on children’s vocabulary development among 50 parent–child dyads. Parent–child interactions at ages 18, 30, and 42 months were analyzed in relation to children’s vocabulary skills one year later at each time point. Controlling for socioeconomic status, input quantity, and children’s previous vocabulary abilities, Rowe found that qualitative aspects of child-directed speech from parents, such as lexical diversity and the use of decontextualized language, significantly explained additional variance in children’s vocabulary gains.

Compared to input, output has received less attention in child language research despite its significance (Ribot, Hoff, and Burridge 2018). Language production allows children to practice newly acquired vocabulary and grammatical structures and solidify their understanding and use of language (Swain 2005). Children can also receive feedback from caregivers and peers, which refines their linguistic skills and helps resolve errors in real-time (Clark 2014). Studies on first language development have shown that children who are more talkative have stronger language skills than their reticent peers (Evans 1996). The frequency and quality of child language output activities, such as storytelling or structured conversations, have also been found to be associated with vocabulary knowledge and narrative skills (Sparks and Reese 2013).

Language input-output is particularly critical in vocabulary development among EB children (Paradis, Genesee, and Crago 2011; Paradis 2023). In a recent study, Daskalaki and colleagues (2024) studied 47 Mandarin-English EB children, ages 7 to 16, in Canada to examine the relationship among parental attitudes, home language use, schooling, and Mandarin language development. The authors found that positive parental attitudes correlated with more Mandarin use at home, and more Mandarin use at home was associated with improved vocabulary and wh-question syntactic accuracy. Among the different school types, Mandarin-English bilingual schools significantly predicted higher Mandarin vocabulary development compared to English-only schools. Research on EB children’s vocabulary development has also consistently shown a strong correlation between L2 input and their vocabulary growth in L2. For example, Hoff et al. (2012) found that among Spanish-English EB toddlers in Florida who are exposed to both Spanish and English at home, children’s relative amount of exposure in each language predicts their rate of vocabulary and grammatical development of each language.

### Emergent bilinguals’ L2 Reading development

Compared to monolingual children, bilingual children navigate between two language systems, which influences their development of phonological awareness, vocabulary, and syntax, all of which are key components of L2 reading. For bilingual children, reading in their L2 involves not only the typical cognitive challenges of reading but also the simultaneous development of oral language in both of their languages. Some bilingual children, particularly EBs from households where a minority language is spoken, encounter the additional challenge of learning to read in L2, a societal language that may not be used at home.

Research has shown that bilingualism influences multiple aspects of reading, primarily due to the shared underlying proficiency between the two languages. According to the *linguistic interdependence hypothesis* (Cummins 1979, 1991), a child’s skills in their first language (L1) play a crucial role in shaping the development of L2 abilities. Bilingual children frequently draw on the linguistic and cognitive resources they have developed in their L1 when reading in their L2. Cross-language transfer is particularly relevant for typologically similar language pairs, such as Spanish and English (Huang et al. 2022; Koda 2008).

Supporting this view, research examining predictors of English reading among Spanish-English EBs show that the relative importance of bilingual language skills may depend on children’s age and the reading construct (e.g. decoding vs. comprehension) (Huang et al. 2022; Proctor, Harring, and Silverman 2017). Huang et al. (2022) investigated the contributions of Spanish and English oral narrative skills to English reading among 95 EB children in early elementary grades from Spanish-speaking homes in the United States (U.S.). All EB children completed a storytelling task, and their narratives were analyzed for linguistic features (number of different new words, lexical diversity, mean length of utterance, and subordination index) as well as discourse-level features. Results from the study revealed that grade level, English lexical diversity, and Spanish subordination index significantly predicted English reading, though the strength of association vary by reading construct. While Spanish subordination index is a stronger predictor than English lexical diversity for the decoding task in English, English lexical diversity was more robust than Spanish subordination index for English reading comprehension.

### Contribution of vocabulary skills to reading

Reading involves a diverse set of skills and knowledge, including linguistic knowledge, metalinguistic awareness, inferencing abilities, and comprehension monitoring (Grabe 2014). Among these, vocabulary knowledge has been a significant focus in existing research. According to the *lexical quality hypothesis* (Perfetti and Hart 2001), the richness of a reader’s mental representation of words, encompassing spelling, pronunciation, and meaning, is essential for reading comprehension. Readers with well-developed lexical representations can recognize words more efficiently, enabling them to grasp word meanings within the text and allocate more cognitive resources to overall text comprehension (Quinn et al. 2015).

Empirical research has provided support for the critical role of vocabulary in reading comprehension. Vocabulary knowledge not only enhances reading comprehension by providing semantic meaning but also supports sentence-level inference. Studies have shown that for EBs, L2 vocabulary knowledge is a strong predictor of their L2 reading comprehension. For example, in a recent longitudinal study of over 100 Spanish-English EB children, Giguere and colleagues (2024) examined the contributions of bilingual oral language and pre-literacy skills of Spanish–English EBs at 5 years of age and their English L2 reading skills at 6, 7, 8, 9, and 10 years. All children in the study were U.S. born, exposed to more Spanish than English at home, and received school instruction in English only. The oral language skill predictors include vocabulary, phonological memory and phonological awareness in both English and Spanish. Bilingual children’s pre-literacy skills, such as English letter recognition and concepts about print were also evaluated. The authors found that EBs’ English vocabulary knowledge and Spanish phonological awareness significantly predicted English reading comprehension.

In another study with Spanish-English EB children across different grades (kindergarten, 3, 5) and instructional contexts (English only vs. bilingual education), Howard and colleagues (2014) used hierarchical regression models to identify the relative importance of socioeconomic status, language and literacy practices, and bilingual vocabulary on Spanish-English EB children’s English reading outcomes. Results from the study showed that, controlling for demographic factors and home and school language and literacy practices, EB children’s English vocabulary remained a significant predictor of English reading accuracy as well as reading comprehension.

While L2 vocabulary’s association with EBs’ L2 reading is well-established, research on the role of EB children’ L1 vocabulary in their L2 reading painted a mixed picture. Some studies have found a unique contribution of EBs’ L1 vocabulary to L2 reading (Miller et al. 2006; Proctor et al. 2006), but others showed that L1 vocabulary did not predict L2 reading comprehension over and beyond L2 vocabulary (Howard et al. 2014; Huang et al. 2022; Proctor, Harring, and Silverman 2017). Howard et al. (2014) found that the results varied by EBs’ grade level. For kindergarteners and 3rd graders, Spanish vocabulary knowledge is a significant predictor of English decoding (kindergarten and grade 3) as well as comprehension (grade 3 only). However, for 5^th^ graders, Spanish vocabulary did not predict either English decoding or comprehension.

These findings suggest that L1 vocabulary may support L2 reading comprehension indirectly by interacting with decoding skills and contributing to cross-linguistic transfer when L2 proficiency is sufficiently developed (Proctor et al. 2006). The pathway from L1 vocabulary to L2 reading may be indirect, whereby strong L1 vocabulary foster the development of L2 vocabulary, which in turn supports L2 reading. Blom et al. (2020) further demonstrated that the extent to which L1 vocabulary supports L2 reading outcomes depends on the typological similarity between the two languages. In their study of bilingual children in the Netherlands, those whose L1 was typologically close to Dutch as the L2 (e.g. a West-Germanic language such as Frisian) had stronger Dutch receptive vocabulary skills than peers whose L1 was more distant (e.g. Turkish), even after controlling for language exposure and socioeconomic status. This particular finding highlights how linguistic similarity can facilitate cross-linguistic transfer, possibly through overlapping conceptual and lexical knowledge. Taken together, these studies show that the contribution of L1 vocabulary to L2 reading varies based on both learner-level factors (e.g. grade level) and language-level factors (e.g. cross-language distance).

To sum up, language input-output appears to be a key driver of EB children’ vocabulary development, which can, in turn, influence their L2 reading outcomes. However, the potential mediation role of bilingual vocabulary knowledge on the relationships between language input-output, and L2 reading development remains unexplored in the extant literature, a gap this study aims to address.

## The Current study

The study investigates whether EB children’s bilingual vocabulary skills in Spanish and English mediate the relationship between their language input-output and English L2 reading skills, including reading comprehension and decoding skills. Note that we use the term *language input-output* to refer specifically to the frequency with which EB children are exposed to and use each language across interpersonal and media contexts. This study first investigated whether Spanish-English EB children’s language input-output (i.e. home English use, school English use, and school Spanish use^1^) differs based on their grade, gender, and parental education. Then, we explored whether children’s bilingual vocabulary skills in Spanish and English mediate the relationship between their language input-output and English reading skills, including reading comprehension and decoding. Specifically, this mediation path model tested (a) how children’s language input-output relates to their English and Spanish vocabulary skills and (b) how children’s English and Spanish vocabulary relate to their English reading comprehension and decoding skills.

## Materials & method

### Participants

Data for the current study were derived from a larger multi-year study that examined Spanish-English EB children’s language and reading development. This study included 338 Latina/o EB children in two elementary grades (1^st^ and 3rd) enrolled in one-way dual language immersion (DLI) programs in a Southwestern city in the U.S.. Participants were from 7 different elementary schools, all of which had a high concentration of Mexican immigrants. In these DLI programs, EBs received both literacy and content area instruction (such as math or science) in both Spanish and English. Per their school records, all participants spoke Spanish at home and were designated as English learners (ELs) at the time of the study. All children were either 1^st^ graders or 3rd graders, and grade levels were roughly balanced in the sample. According to the school report, none of the children had significant speech, language, or developmental disorders. There was no overlap between the children assessed in 2019 and those included in the 2021 sample.

### Procedure

This multi-year study was conducted during academic years from 2019 to 2022. All child participants were recruited with the help of their teachers and staff at school. Year 1 (Fall 2019) data collection took place pre-pandemic during fully in-person instruction. Year 2 (Fall 2020) occurred during the pandemic, with most students experiencing remote or hybrid learning. Year 3 (Fall 2021) saw a return to mostly in-person instruction, though disruptions like student absences and occasional remote learning remained. In Years 1 and 3, trained research assistants conducted in-person assessments at the students’ schools whereas Year 2′s data were collected remotely. Child participants received the language and reading assessments in both Spanish and English in two separate times scheduled at least four days apart. The order of the two testing sessions was also counter balanced. Combined with the lapse of time between the two sessions and the counter-balanced design, we minimized cross-language influence and practice effects. In each session, a native or heritage speaker research assistant of the target language (either English or Spanish) provided directions in the target language of the testing session. The research assistant also administered the child survey at the end of the Spanish session, though the language of the interview varied by child based on their preference. Participants were given multiple short breaks in each session. Due to the school closure in Year 2 (2020–2021), all data were collected remotely via Zoom. The same protocol and procedure were followed except for the change in testing modality (i.e. in person vs. videoconferencing).

All children received a $20 gift card for their participation. The teacher surveys and parent surveys were distributed to the teachers and parents of the child participants via email and collected after all child assessments were completed.

### Instruments

#### English reading measures

We used three subtests in the Woodcock-Johnson IV Tests of Achievement (WJ-IV ACH; Schrank, Mather, and McGrew 2014a) to evaluate English reading skills: (a) the Letter Word Identification (LWI, 78 items), (b) the Word Attack (WA, 32 items), and (c) Passage Comprehension (PC, 52 items). The LWI subtest evaluated the ability to read isolated printed English letters and words, and the WA subtest assessed the ability to read unfamiliar or nonwords (English word decoding). Given the correlation between LWI and WA was very high (*r* = .89), LWI and WA raw scores were averaged and used as an observed variable (hereafter referred to as Decoding) in the path model. The PC subtest measures English reading comprehension skills. The task used a modified cloze procedure and required participants to provide a word that is appropriate in the context of a short passage. For the target grade levels, the publisher-reported reliabilities range from 0.94 to 0.98 for the LWI subtest, 0.92 to 0.96 for the WA subtest, and 0.87 to 0.93 for the PC subtest.

#### English and Spanish vocabulary measures

To assess English and Spanish vocabulary knowledge, we used the English vocabulary subtest (Test 1: Picture Vocabulary) and the Spanish equivalent (Test 10: Vocabulario sobre dibujos), respectively, in the Woodcock-Johnson IV Tests of Oral Language (WJ-IV OL; Schrank, Mather, and McGrew 2014b). Both subtests required the participants to identify and name the object presented in a picture. Although the constructs in both languages are equivalent, the items in the two versions are not direct translations. The publisher-reported reliability coefficient ranges from .77 to .94 for both the English and Spanish subtests.

#### Language input-output surveys

To collect information about child participants’ demographic backgrounds and language input-output at home and in school, we administered a Child Survey and a Teacher Survey, using questions adapted from previous studies (Huang et al. 2020). The Child Survey included questions about the participants’ demographic information (e.g. home language), their language use (e.g. How frequently do you use English at breakfast in the morning?), and frequency of reading various genres in English and in Spanish (e.g. How often do you read magazines in English/Spanish outside of school?) on a Likert type scale. To create an ‘*English use at home*’ variable, we calculated a composite score by averaging child-reported ratings across three survey items that asked how often they use English use when they get home, during the weekend, and when watching media using a 5-point Likert scale (1 = no English; 2 = a little English; 3 = some English; 4 = mostly English; 5 = only English).

The Teacher Survey provided information about child participants’ frequency of use of Spanish and English with the teacher, with their peers, and with other adults in school (e.g. How often does the child speak with you, the teacher, in Spanish/English?) on a Likert response scale (0 = Never; 1 = Rarely; 2 = Sometimes; 3 = Very often; 4 = Usually; 5 = Always). Teachers who responded ‘Don’t know’ were treated as missing. There were three questions for each language, totaling 6 questions about child participants’ language use in school. We created a composite score for ‘*English use at school*’ and ‘*Spanish use at school*’ by averaging teacher ratings across three questions that asked about children’s use of English or Spanish at school, respectively.

Although we measured both language input (exposure) and language output (use), our survey items focused more on output. We thus labeled the composite variables as ‘*English use at home*,’ ‘*English use at school*,’ and ‘*Spanish use at school*’ for brevity and consistency.

#### Demographic surveys

Parents reported their demographic information (e.g. education level) and home language practices on a Likert type scale, using questions adapted from previous studies (e.g. Huang et al. 2020). Due to the high missing rate in the parent survey, we only used the parent education level variable from the Parent Survey. The Child Survey also included questions about children’s demographic information, such as date of birth.

### Data analysis

Analyses were conducted using Mplus version 8.8 (Muthén and Muthén 1998–2022) with CLUSTER command with the ‘complex’ method was used to account for several children clustered within the same teacher in a given year. First, a series of multivariate regression analyses were employed to explore differences in children’s language input-output (i.e. home English use, school English use, and school Spanish use) based on their grades, sex, and parental education, separately. Then, path analysis was conducted to examine whether children’s oral language skills (i.e. vocabulary skills in Spanish and English) mediate the association between their language input-output (i.e. home English use, School English use, and School Spanish use) and English reading skills (i.e. reading comprehension and decoding skills). As mentioned previously, a latent factor was created for reading skills given the high correlation between reading comprehension and decoding skills (*r* = .89, *p* < .001). Model fit was evaluated using root mean square error of approximation (RMSEA), Comparative Fit Index (CFI), Tucker-Lewis index (TLI), and Standardized Root Mean Square Residual (SRMR). RMSEA values within the range of 0.05 and 0.08 are deemed acceptable (Fabrigar et al. 1999), CFI and TLI values above .95 indicate an excellent fit, and a SRMR value below .08 is considered indicative of a good fit (Hu and Bentler 1999). To identify control variables, we ran correlations among the key variables (i.e. language input-output, oral language skills, and reading skills) and variables potentially associated with them, including child sex, grade level (1^st^ vs. 3rd), parent education, and the year of data collection (Year 1, 2, and 3). No variable other than grade level was significantly related to key variables in the model, and thus, grade level was included as a control variable in the analysis. Missing data was handled using full information maximum likelihood (FIML).

## Results

### Sample characteristics

Table 1 presents descriptive statistics of study variables for total sample and by Grade. Grade level (1^st^ vs 3rd) and sex (boys vs girls) are balanced in the sample. Nearly half of the participants were recruited in Year 3 of the study (47.63% overall; 44.19% of 1^st^ graders; 51.20% of 3rd graders), followed by Year 1 (28.11%) and Year 2 (24.26%). Among the parents who provided the information (*n* = 170 out of 338), over half reported that their highest education was high school (52.35% overall; 51.16% of 1^st^ graders; 53.57% of 3rd graders), followed by associate degree/college degree (24.71%), some college (12.94%), and graduate degree (10%). On average, participating children reported using nearly ‘3 = some’ English at home (*M* = 2.81; *SD* = 1.09), with 1^st^ graders showed a lower average score (*M* = 2.67, *SD* = 1.14) than 3rd graders (*M* = 2.95, *SD* = 1.02; *t*(330) = −2.34, *p* = .02). Teachers reported that these participating children used both English (*M* = 3.12; *SD* = 1.46) and Spanish (*M* = 3.05; *SD* = 1.44) very often (= 3) at school. No grade difference was found on English use at school between 1^st^ graders (*M* = 3.27; *SD* = 1.56) and 3rd graders (*M* = 2.98, *SD* = 1.34; *t*(314) = 1.76, *p* = .08), but 1^st^ graders used less Spanish at school (*M* = 2.86, *SD* = 1.60) compared to 3rd Graders (*M* = 3.23, *SD* = 1.26; *t*(305) = −2.28, *p* = .02). Compared to the 1^st^ graders, 3rd graders scored higher in both Spanish and English language measures (English Vocabulary: *t*(318) = −10.23, *p* < .001; Spanish Vocabulary: *t*(327) = −2.71, *p* = .007) as well as English reading measures (Reading Comprehension: *t*(321) = −16.69, *p* < .001; Decoding: *t*(321) = −18.40, *p* < .001).

### Differences in language input-output by child characteristics

The three indicators of children’s language input-output were compared using separate multivariate regression models based on grade level, sex, and parent education (Table 2). Compared to the 1^st^ graders, 3rd graders used more English at home (*β* = .13, *p* < .05), but no level difference was found in their English nor Spanish use at school (*β* = −.11, *p* > .05 and *β* = .13, *p* > .05, respectively). Boys and girls did not differ in their use of English at home (*β* = .10, *p* > .05), English at school (*β* = −.10, *p* > .05), and Spanish at school (*β* = .02, *p* > .05). Parent education level was not associated with in EB children’s use of English at home (*β* = .02, *p* > .05), English at school (*β* = .10, *p* > .05), and Spanish at school (*β* = −.15, *p* > .05).

### Path analysis: mediation effect of vocabulary skills

**Figure 1.**
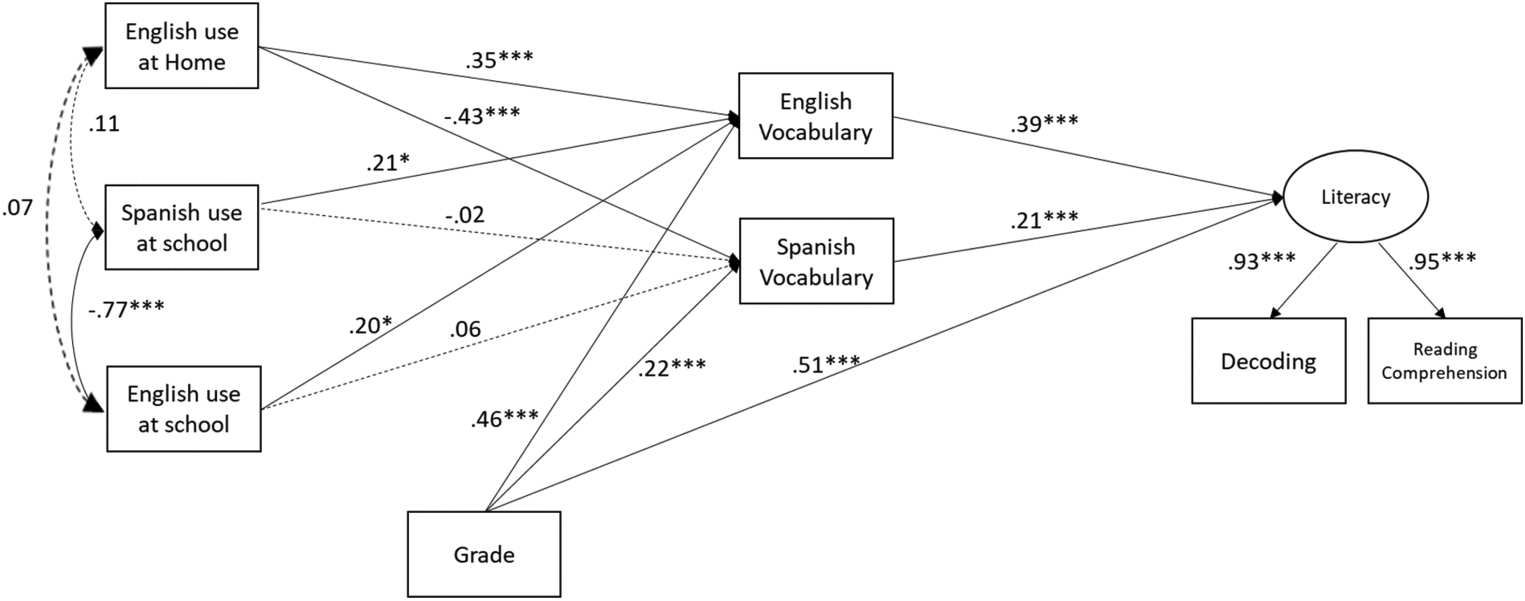
Standardized coefficients (*β*) in path model between language use and literacy skills (*N* = 338). Note. Solid lines indicate a significant path at a *p*-value of ≤ 0.05, while dotted lines indicate a non-significant path. *** *p*<.001; ** *p* < .01; * *p* < .05. A figure of the path model indicating the pathways from language input-output (English use at home, Spanish use at school, English use at school) to English reading (Decoding and Reading comprehension), with English vocabulary and Spanish vocabulary as mediators.

Figure 1 presents the parameter estimates from the path model, illustrating the mediation effects of EB children’s English and Spanish vocabulary skills on the association between their language input-output and reading outcomes. The mediation path model was an acceptable fit to the data: RMSEA = .07, TLI = 0.97, CFI = 0.99, and SRMR = .05. Results from the model showed that English reading skills were positively predicted by vocabulary skills in English (*β* = .39; *p* < .001) as well as in Spanish (*β* = .21; *p* < .001). Further, English vocabulary skills were positively predicted by EB children’s English use at home (*β* = .35; *p* < .001), Spanish use in school (*β* = .21; *p* < .05) and English use in school (*β* = .20; *p* < .05). Spanish vocabulary skills were negatively predicted by EB children’s English use at home (*β* = −.43; *p* < .001), but neither English nor Spanish use at school significantly predicted Spanish vocabulary skills. Third graders scored higher in English vocabulary, Spanish vocabulary, and reading skills than 1^st^ graders.

Results for the indirect effect of vocabulary skills indicated that the association between EB children’s home English use and their English reading skills were positively mediated by English vocabulary skills (*β* = .14; *p* < .001) but negatively mediated by Spanish vocabulary skills (*β* = −.09; *p* < .001), counteracting the total indirect effect from children’s home English use and English reading skills (*β* = .05; *p* > .05). It was further found that children’s higher Spanish use in school preidcted higher English reading skills via advanced English vocabulary skills (*β* = .08; *p* < .05).

## Discussion

The aims of the current study are twofold: to explore whether Spanish-English EB children’s language input-output differs based on grade, sex, and parental education and to examine the relationships between language input-output, Spanish/English vocabulary skills and English reading skills (i.e. reading comprehension and decoding). Our sample consisted of exclusively Spanish-English Latina/o EBs in DLI programs who receive language and content instruction in both languages. Expanding on the predominant research on EBs in mainstream English-only programs, this study focused on EBs in DLI programs, a learning context where EBs’ development has been limitedly studied. The results showed that EB children’s language use at home differed by grade levels, but not by sex or parent education. English and Spanish use at school did not differ by grade level, sex, or parent education. Results from the path analyses provided empirical support for our proposed model, indicating Spanish and English vocabulary skills as mediators in the association between children’s language use and reading outcomes.

### Variability in bilingual children’s language input-output by grade level

Results from this study showed that EB children in DLI programs use both Spanish and English in school (3 = very often), at home (nearly 3 = some), and in various other contexts reported by EB children themselves and by their teachers. Multivariate regression analysis of teacher reports also revealed no grade-level differences in EBs’ Spanish and English use in school, suggesting that the children in this study’s DLI programs may benefit from the program by continuing to use and practice both of their languages in the school context.

Their language input-output at home, however, painted a different picture. Third graders reported more English input-output at home than 1^st^ graders, which possibly indicate some decline in Spanish input-output at home among 3rd graders. The results may indicate that some degree of language input-output shift is potentially inevitable even for bilinguals in DLI programs that aim to promote bilingual proficiency and biliteracy and actively maintain bilingual language use in the school context. The results may also be attributed to 3rd graders’ higher English proficiency: 3rd graders performed significantly better than 1^st^ graders in English and Spanish vocabulary and English reading measures. It is worth noting that while 3rd graders’ higher English vocabulary and English reading scores are not surprising in the U.S. context, their higher performance on Spanish vocabulary measures compared to 1^st^ graders contrasted previous studies showing stagnation and even regression of Spanish skills among Latino/a children in the U.S. after age 5 (e.g. Bedore et al. 2016; Proctor et al. 2010; Silva-Corvalán 2014). This result may reflect the positive effect of the DLI program on developing EB children’s L1 skills.

Alternatively, compared to 1^st^ graders, 3rd graders may exhibit a stronger preference for using English over Spanish at home. Research indicates that language preference is fluid, influenced by context (Caldas and Caron-Caldas 2002), the amount and quality of input (Juan-Garau and Pérez-Vidal 2001), and cultural perceptions (Dewaele 2011). While Spanish is the language of instruction at school, which may reinforce its use, EBs have more freedom in their language choices at home. Given their higher English proficiency, 3rd graders may feel more comfortable using English in the home context.

### Relationships among language input-output, Spanish/English vocabulary knowledge, and English reading

We expected to find a strong relationship between language input-output and their vocabulary knowledge in both English and Spanish. The prediction was supported by our data. For English vocabulary, the results showed that EB children’s language input-output, including English use at home, English use in school, and Spanish use in school, positively predicted English vocabulary knowledge. The results corroborated previous research that showed a positive correlation between EBs’ English language use and their English language proficiency (e.g. Paradis, Genesee, and Crago 2011; Paradis 2023). Notably, EB children’s higher Spanish use in school was also positively correlated with more advanced English vocabulary knowledge, suggesting that L1 use (i.e. Spanish in this case) could contribute to EBs’ learning of English as a L2.

In contrast, the only significant predictor of Spanish vocabulary was English use at home, and this association was negative. This finding align with some previous studies with toddler- or preschool-aged EB population, such as Hoff and colleagues’ study (2012) on Spanish-English EBs in Florida, USA or Scheele and colleagues’ (2010) work with Moroccan–Dutch and Turkish–Dutch bilinguals in the Netherlands. In a study with preschool-aged EBs in Norway, Rydland and Grøver (2021) found home literacy environment, operationalized as numbers of L1 and L2 books in the home, to be more important in accounting for differences in L1 vocabulary skills than language use. Future research examining the contributions of and interactions among the multiple factors in EB children’s language development would further clarify the relationships between language input-output and EB children’s L1 vocabulary knowledge.

With regard to the relationships between bilingual vocabulary and English reading outcomes, we also predicted that both EB children’s English and Spanish vocabulary would contribute to English decoding and English reading comprehension. Our findings supported these predictions. The significant role of English oral language in English decoding and reading comprehension replicated previous results (e.g. Huang et al. 2022; Proctor et al. 2012; Silverman et al. 2015). Notably, Spanish vocabulary also showed a significant contribution to English reading, a finding that contradicted a recent study with Spanish-English EBs by Giguere and colleagues (2024). Given that Giguere and Hoff’s (2024) focused on Spanish-English EB children who received instruction in English only, it is presumed that their sample had lower Spanish proficiency than the current sample. The differences in the two studies’ sample characteristics may account for the divergent results. Our findings provide support for the SVR model, which conceptualizes reading comprehension as a function of both decoding and language comprehension. In this study, English vocabulary, which is a key component of language comprehension was a significant predictor of both English decoding and reading comprehension. This finding also demonstrates that, even for EB children with relatively high Spanish language use, English vocabulary remains a strong predictor for English reading. The current study contributes to the field where very limited research has examined language-reading associations in bilinguals in DLI programs, which provide formal instruction in both English and the children’s L1.

The results indicated that English reading, encompassing both decoding and comprehension, was significantly predicted by EBs’ L1 (Spanish) vocabulary (*β* = .21) and L2 (English) vocabulary (*β* = .39). We interpreted our results as providing support for cross-linguistic transfers between L1 proficiency to L2 reading, corroborating the work of Miller et al. (2006) and Huang et al. (2022). Given that Spanish and English are typologically close and share cognate words, researchers have argued for potential cross-linguistic transfers, especially when learners receive instruction in both languages (Proctor et al. 2010). It is worth noting that both previous studies by Miller et al. and Author et al. targeted EB children receiving instruction in their L1 Spanish as well as English; EB participants in Author et al. were also enrolled in one-way DLI programs and the bilingual sample in Miller et al. received some Spanish language and literacy support. It is worth noting that the current results showed that the transfer effects are robust and can be observed across different language measures of oral language skills. Whereas both previous studies (Huang et al. 2022; Miller et al. 2006) used a narrative elicitation task and language sample analysis, the vocabulary measures in the current study were standardized assessment. Researchers have argued that narrative elicitation tasks are more appropriate for EBs from culturally diverse backgrounds, particularly for evaluating language proficiencies across languages (Miller et al. 2006). Future research that compares the validity inferences for both standardized and narrative elicitation measures and their relationships with EBs’ L2 reading would help clarify the role of language measures in bilingual research.

Finally, the current results partially supported our predictions about the indirect effects of language input-output on EB children’s English reading (see Figure 1). First, children who reported using more English at home had more advanced English vocabulary skills and elevated English reading. Their English reading skills were positively mediated by English vocabulary skills, revealing a positive indirect effect of home language use on English reading outcome. However, EB children’s English use at home and English reading outcomes were negatively mediated by Spanish vocabulary, thus counteracting the total indirect effect of language use at home on English reading. Additionally, EB children who use more Spanish at school have more advanced English vocabulary skills, which in turn predicted elevated English reading. This particular finding suggested that the Spanish language use in school had an indirect effect on English reading, which is mediated by English vocabulary. As the first study to explore how Spanish/English vocabulary mediates EB bilingual children’s language use and English reading, our findings lack direct reference points for comparison. Nevertheless, our results lay a foundation for future research to refine the model and develop a comprehensive theory to explain the interplay between EB bilingual children’s language use, L1 and L2 vocabulary, and English reading.

## Limitations

Limitations of the study must be acknowledged. First, the present study defined and operationalized ‘language input-output’ as the exposure to and frequency of using the target languages. Similar to many previous research studies (e.g. De Houwer 2007; Jia, Aaronson, and Wu 2002), we used children’s self-report for home language input-output and teacher report for children’s language input-output in school. Although frequencies of language input-output could be valid indications of quantity, they may not tap into the quality and richness of language input-output, which is defined in the literature as the interlocutors’ target language proficiency, the richness of EBs’ experience with extra-curricular activities, etc., and has been found to better predict language outcomes than quantity (e.g. Hirsh-Pasek et al. 2015). Future investigations that consider both quantity and quality are clearly needed to specify the role of language input-output in EB children’s language development. Relatedly, the study did not include a direct measure of Spanish language use at home. Although all participants were Spanish–English bilinguals, only child-reported English use at home was collected. These data allowed us to infer relative Spanish exposure indirectly, but the lack of a parallel measure for Spanish limits our ability to fully characterize bilingual language input-output. The study’s reliance on EBs’ self-report and teacher report can also limit the validity of the inferences to provide accurate reports of language input-output. Future research may incorporate objective measures such as classroom observations or consider using the experience sampling method (Arndt, Granfeldt, and Gullberg 2023). Because language learning is a complex topic, research that examines other predictors of bilinguals’ language development, such as EBs’ working memory and home literacy environment would help provide a more comprehensive picture of the factors that predict EBs’ language outcomes (e.g. Farnia and Geva 2011; Swanson, Orosco, and Lussier 2015).

Second, the current study did not collect information about family language patterns or parents’ countries of origin. Research has shown that family language patterns – specifically, which languages are spoken and by whom at home - are closely associated with the quantity and quality of language input that bilingual children received (Verhagen, Kuiken, and Andringa 2022). In addition, Mora and Lopez (2023) found that U.S. Latinos of Central American origin, compared to their Mexican and Puerto Rican peers, placed greater emphasis on the importance of maintaining Spanish proficiency among future Hispanic generations. Future research should take into consideration both family language patterns and parental country of origin to provide a comprehensive account of EB children’s language input-output.

Third, because of the cross-sectional design, our results relating to grade-level differences in language input-output, Spanish/English vocabulary knowledge, and English reading skills should be interpreted with a grain of salt. What the study found were grade-level differences, not developmental changes. Future studies that utilize a longitudinal design following the same children over time would afford us the opportunity to examine developmental changes as well as changes in the relationships among these variables as children move through early elementary grades.

Additionally, the current study is pragmatically driven to focus on English reading outcomes because of the education accountability in English reading and the importance of English reading in academic achievements. As many Spanish-English EBs, especially those in DLI programs, are also literate in Spanish, examining the relationships between their bilingual oral proficiencies and biliteracy skills would help unveil EB children’s developmental pathways from language to literacy.

Finally, it is important to acknowledge that the COVID-19 pandemic introduced considerable variability in students’ learning experiences across the three years of the study. Differences in instructional modality (e.g. in-person, remote, hybrid), along with disruptions such as student absences, inconsistent access to technology, limited familiarity with remote learning environments, and reduced opportunities for classroom interaction, may have influenced EB children’s language and reading development outcomes in ways that were not fully captured in our analyses.

## Conclusion and implications

To conclude, this study extended the literature by testing our hypothesis that EB children’s language input-output is associated with English reading outcomes through advancements in both English and Spanish vocabulary. Building on existing research on EB children in mainstream English-only programs, this study focused on EB children in DLI programs, a learning context where bilinguals’ development has been less explored. Our findings reveal differences in language input-output by grade level; specifically, a potential increase in English use at home for older children (3rd graders compared to 1^st^ graders) was observed. This may indicate that some degree of language use shift is likely unavoidable, even for EB children in DLI programs. However, we also found evidence that older children (3rd graders) continue to use both English and Spanish in the DLI program context, highlighting DLI program’s significant role in maintaining the minority home language. Our findings also support the robust role of both L1 and L2 input-output in English vocabulary development, which was positively associated with advanced English reading outcomes. EB children’s English use at home and Spanish use at school predicted their English vocabulary skills, which were linked to English reading skills. These results demonstrated the additive, rather than subtractive role of EBs’ L1 use in their L2 (English) reading. Findings from our study also highlight the associations between vocabulary and English reading, underscoring the importance of developing EBs’ vocabulary proficiency to support their English reading success (Petscher et al. 2020).

These findings have important implications for instructional practices and education policy for EB children. To support balanced bilingual and biliteracy development over the long term, it is crucial to address the role of language input-output in EBs’ language and reading development. Families and educators should work together to create balanced linguistic and biliteracy environments. such as by enrolling children in DLI programs. Promoting bilingualism and biliteracy is a central goal of DLI programs (Howard et al. 2018). In English-dominant educational systems, EB children often face pressure to prioritize English, which can lead to a decline in their home language use. Research shows that well-implemented DLI programs have the potential to counteract inequitable outcomes compared to monolingual or subtractive models (Dorner and Cervantes-Soon 2020). By valuing and building on students’ existing language knowledge, DLI programs help foster continued development in both languages.

Additionally, given the minority status of Spanish in the U.S. context, strategies to encourage EBs to use Spanish more frequently, especially at home, should be explored and implemented. For educators, prioritizing vocabulary development is essential to enhance EBs’ foundational language skills and improve their L2 reading outcomes. This can involve explicit vocabulary instruction and fostering opportunities for meaningful, context-rich language input-output across various subject areas. Furthermore, professional development programs should focus on equipping educators with evidence-based practices that address oral language and vocabulary development. By doing so, educators will be better prepared to meet the diverse linguistic needs of EBs and support their language and reading success.

To our knowledge, this is the first study that investigated the relationships among language input-output, Spanish/English vocabulary skills, and English reading among Spanish-English EB children in DLI programs. The study contributes to the field by specifying the connections among the three constructs, highlighting significant relationships between language input-output and vocabulary as well as between bilingual vocabulary skills and English reading. In particular, this study identified vocabulary skills as a mediator in the pathways linking language input-output to EB children’s L2 reading outcomes. The present study also made a novel contribution to the field by extending the results from EB children in English-only programs to those in DLI programs, who receive more balanced language input-output and formal instruction in both English and Spanish. These findings deepen our understanding of bilingual language development and provide insights into supporting EB children’s reading success in dual-language learning contexts.

## Figures and Tables

**Table 1.** Descriptive statistics of key variables and participant characteristics (N = 338)

Variable name	Total				Grade 1 (50.89%)				Grade 3 (49.11%)			
	N	Mean/%	SD	Range	N	Mean/%	SD	Range	N	Mean/%	SD	Range
English Reading skills (Raw score)												
Reading comprehension	323	17.18	7.84	0–33	162	11.86	5.46	4–25	161	22.53	6.02	0–33
Decoding	323	35.79	17.59	2–69	162	23.25	12.21	2–54	161	48.41	12.36	6–69
Vocabulary skills (Raw score)												
English vocabulary	320	19.45	5.15	4–31	160	16.89	4.97	4–27	160	22.01	3.90	9–31
Spanish vocabulary	329	22.05	5.74	6–35	165	21.21	5.48	7–32	164	22.91	5.89	6–35
Language input-output												
English use at home	332	2.81	1.09	1–5	169	2.67	1.14	1–5	163	2.95	1.02	1–5
English use at school	316	3.12	1.46	0–5	156	3.27	1.56	0–5	160	2.98	1.34	0–5
Spanish use at school	307	3.05	1.44	0–5	149	2.86	1.60	0–5	158	3.23	1.26	0–5
Sex	338				172				166			
Girl		50.59%				54.07%				46.99%		
Boy		49.41%				45.93%				53.01%		
Study Year	338				172				166			
Year 1: 2019–20		28.11%				30.81%				25.30%		
Year 2: 2020–21		24.26%				25.00%				23.49%		
Year 3: 2021–22		47.63%				44.19%				51.20%		
Parent education	170				86				84			
High school (= 1)		52.35%				51.16%				53.57%		
Some college (= 2)		12.94%				9.30%				16.67%		
Associate /College degree (= 3)		24.71%				29.07%				20.24%		
Graduate degree (= 4)		10.00%				10.47%				9.52%		
Mean		1.92	1.08	1–4		1.99	1.11	1–4		1.86	1.05	1–4

Note. The maximum possible scores are as follows: Reading Comprehension (52), Decoding (78), English Vocabulary (54), and Spanish Vocabulary (53).

**Table 2.** Differences in language input-output by grade, child sex, and parent education.

Variables	English use at home			English use at school			Spanish use at school		
	*β*	(SE)	*p*	*β*	(SE)	*p*	*β*	(SE)	*p*
Model 1									
Grade (1 = 3rd grader)	0.13	(0.05)	0.02	−0.11	(0.12)	0.35	0.13	(0.12)	0.30
Model 2									
Sex (1 = boy)	0.10	(0.06)	0.08	−0.10	(0.07)	0.15	0.02	(0.07)	0.74
Model 3									
Parent education	0.02	(0.11)	0.84	0.10	(0.11)	0.36	−0.15	(0.12)	0.18

Note. Each child characteristic was analyzed individually.

* *p* < .05

## Footnotes

1. Note that our study measured both language input (exposure) and output (use/production), but we labeled the variables as ‘English use at home,’ ‘English use at school,’ and ‘Spanish use at school’ for consistency and brevity.
